# Prevalence and Risk Factors of Diabetes Mellitus: A Community-Based WHO STEPwise Approach to Surveillance (STEPS) Survey in Rural Haryana, India

**DOI:** 10.7759/cureus.89089

**Published:** 2025-07-30

**Authors:** Jaya Kumari, Shalini Ray, Sunil Gupta

**Affiliations:** 1 Community Medicine, Prasad Institute of Medical Sciences, Lucknow, IND; 2 Community Medicine, PES University Institute of Medical Sciences, Bangalore, IND; 3 Orthopedics, Naraina Medical College and Research Center, Kanpur, IND

**Keywords:** “diabetes mellitus”, non-communicable disease (ncd), prevalence study, steps survey, type 2 diabetes

## Abstract

Background: Non-communicable diseases (NCDs), including type 2 diabetes mellitus, are emerging as major health concerns across India. Diabetes, once considered a predominantly urban condition, is increasingly being reported from rural populations as well, reflecting broader lifestyle and epidemiological transitions. National reports highlight a growing prevalence of high blood glucose levels, particularly among adults, with a rising trend in both awareness and disease burden. India is now among the countries with the highest number of individuals living with diabetes, necessitating urgent public health attention. This study examines the prevalence of type 2 diabetes and its associated risk factors among adults in a rural area of Haryana, contributing to the evidence base required for targeted NCD prevention and control strategies in similar settings.

Materials and methods: The study was conducted among 910 adults aged ≥18 years from villages under the Primary Health Center (PHC) Garhi Harsaru, Haryana, India. Cluster random sampling, using probability proportional to size (PPS), was employed to recruit study participants. All the study participants were subjected to the WHO STEPS questionnaire, and random blood glucose estimation was done. Apart from these, demographic, socioeconomic, and anthropometric details, along with blood pressure and physical activity, were recorded, and their association with the prevalence of diabetes was studied. Statistical analysis was done using SPSS version 26.0 (Chicago, IL: IBM Corp.). Pearson's chi-squared test was used to evaluate differences between groups for categorical variables. Multivariate logistic regression was used to examine the independent associations.

Results: The prevalence of newly detected diabetes was 6.2%. Adults aged 50-59 years (OR: 3.4), overweight, and obesity (OR: 5.80) were found to be the independent risk factors significantly associated with diabetes mellitus in the study population.

Conclusions: The prevalence of diabetes is rising, even in the rural population of Haryana. Targeted preventive strategies focusing on lifestyle modification and education should be prioritized in primary care settings to address this growing rural health challenge.

## Introduction

Non-communicable diseases (NCDs) are a major public health challenge worldwide due to their associated morbidity and mortality. NCDs were initially considered diseases of wealthier countries; however, they are now prevalent in all socioeconomic strata in society, including both rural and urban areas of developing nations. In India, NCDs accounted for 61.8% of total deaths in 2017 [1]. Globally, more than 60% of the economic burden in 2017 was attributed to NCDs, highlighting their significant impact on healthcare systems.

Among the major NCDs, diabetes mellitus has emerged as a significant public health issue. The number of diabetes cases in India increased markedly from 162.7 per one lakh population in 1990 to 264.5 per one lakh in 2021, as reported by the Global Burden of Disease Study (GBDS) 2021 [2]. According to the National Family Health Survey (NFHS-5, 2019-21), approximately 15.4% of women and 16.8% of men in India exhibit high or chronically elevated blood sugar levels [3]. High BMI (overweight or obesity) is a key modifiable risk factor contributing to this epidemic. The prevalence of overweight among Indian adults rose from 9.0% in 1990 to 20.4% in 2016. As per GBDS 2021, the prevalence of diabetes is 52.2% among overweight adults aged over 20 years, compared to the global average of 19% [2]. NFHS data also indicate that 41% of women and 44% of men in India are obese, placing them at heightened risk for developing NCDs, including diabetes [3].

Given the epidemiological transition of diabetes in India, particularly its increasing burden in rural populations, there is a critical need to identify risk factors and high-risk groups for community-based prevention strategies. However, there remains a paucity of data on the prevalence and determinants of diabetes in rural areas of Haryana. With this background, the present study was conducted among a cross-sectional sample of 910 individuals, selected through cluster random sampling in a rural Primary Health Centre (PHC) area of Haryana, to estimate the prevalence of type 2 diabetes mellitus (T2DM) and identify its associated risk factors.

## Materials and methods

This study was conducted over a two-year period in the villages under the PHC Garhi Harsaru, located in the Gurugram district of Haryana. There are a total of 14 villages covered under this PHC, which adds up to a population of 45,729. The study was conducted among adults aged 18-59 years, residing in the study area for at least six months. Adults aged 18 years or older who were already diagnosed with non-communicable diseases and eligible residents who could not be contacted, despite three successive visits, were excluded from the study.

Considering a 30% prevalence of lifestyle-related risk factors for non-communicable diseases among the adult population, and using a relative error of 10% at a 95% confidence interval [4], the sample size was calculated using the formula: $N = \frac{1.96 \times 1.96 \times 30 \times 70}{9}$ = 896.37, which was taken as the optimum sample size [5]. Cluster random sampling, using the probability proportional to size (PPS) method, was employed as the sampling method. In the present study, all 14 villages of the PHC were taken as natural clusters. The villages were chosen as primary sampling units (PSU) and the eligible adults (in the households of the villages) as secondary sampling units (SSU). As shown in Table 1, all 14 villages (column A) with their population (column B) were listed. All the households in the cluster were assigned serial numbers. The first household for each cluster was chosen randomly. Starting from the first selected household, a house-to-house survey was done, and eligible subjects fulfilling the inclusion criteria from each household were included in the study. A total of 65 study participants were included from each cluster to reach the final sample size of 910.

**Table 1:** List of villages in Primary Health Centre (PHC) Garhi Harsaru.

S. no.	Name of village (column A)	Population (column B)	Cumulative population (column C)	Clusters identified
1.	Bamdoli	763	763	1st cluster
2.	GarouliKallan	1529	2292	
3.	Hayatpur	1936	4228	2nd cluster
4.	KherkiMajra	1958	6186	
5.	Chandu	2165	8351	3rd cluster
6.	Sadrana	2260	10,611	4th cluster
7.	Badha	2478	13,089	
8.	Harsaru	2976	16,065	5th cluster
9.	GarouliKhurd	3212	19,277	6th cluster
10.	Budera	3470	22,747	7th cluster
11.	Kadipur	4190	26,937	8th cluster
12.	Dhankot	5243	32,180	9th, 10th cluster
13.	Basai	5546	37,726	11th cluster
14.	GarhiHarsaru	8003	45,729	12th, 13th, 14th cluster

The primary study tool was a pre-tested, semi-structured questionnaire and a modified, pre-tested WHO STEPwise Approach to Surveillance (STEPS) instrument [6]. WHO's STEPS approach is the WHO’s recommended tool for assessment of chronic or non-communicable diseases and their risk factors. Sociodemographic details, history of comorbidities, physical activity, family history of NCDs, history of tobacco and alcohol consumption were elicited. Study participants underwent anthropometric assessment and blood pressure measurement; a random blood sugar test was conducted for those who consented. Random blood glucose estimation was conducted for only 895 participants who consented to it. All individuals with >200 mg/dL random blood glucose were subjected to further biochemical tests for confirmation at the nearest health facility. Approval from the institutional ethics committee was obtained prior to conducting the study. The different operational definitions used in the study are defined further below.

Current tobacco consumers were those who were currently smoking or chewing any form of tobacco daily. Current alcohol consumers were those who had consumed alcohol in the past week. For the assessment of physical activity, respondents were asked whether they engaged in any specific physical activity for at least 30 minutes during the day. According to CDC guidelines, individuals who engage in moderate physical activity, such as walking/sports, for 30 minutes a day, at least five days a week, are considered physically active [7]. BMI was classified according to the WHO classification for Asians [8]. Random blood glucose was measured using a Freestyle Optium Glucometer (Chicago, IL: Abbott Laboratories) (glucometer was standardized by cross-checking laboratory results). Individuals in whom glucometer random blood sugar (GRBS) was ≥200 mg/dL were considered to have diabetes according to the American Diabetes Association (ADA) classification and were referred for a confirmatory test at the nearest health facility [9]. Hypertension was classified according to the Eighth Joint National Committee (JNC 8) classification. According to JNC 8 guidelines, stage I hypertension is defined as systolic blood pressure (SBP) ≥140-159 mmHg or diastolic blood pressure (DBP) ≥90-99 mmHg, whereas stage II hypertension is defined as systolic blood pressure (SBP) ≥160 mmHg or diastolic blood pressure (DBP) ≥100 mmHg.

The collected data, after proper coding and cleaning for possible errors, were entered in the MS Excel (Redmond, WA: Microsoft) spreadsheet. Analysis was carried out using SPSS version 26.0 (Chicago, IL: IBM Corp.) for Windows. Categorical data were presented as percentages. Pearson’s chi-square test was used to evaluate differences between groups for categorized variables. A significant p-value was considered when it was <0.05, and it was considered highly significant when the p-value was ≤0.01. Each respondent was assigned a code number to maintain their confidentiality. Logistic regression analysis was performed to identify factors significantly associated with risk.

## Results

Table 2 presents the sociodemographic and behavioral characteristics of the study participants. Out of the total study sample of 910 participants, the majority were females, at 60.2%. Approximately 48.3% of the study participants belonged to the 30-49 years age group. About 75.5% of participants were married, with a maximum of 32.2% of participants completing their primary schooling, about 46.7% of the participants were homemakers, and about 53.4% belonged to upper middle socioeconomic status (SES), followed by 23% to middle class, and 4.5% to lower SES. The prevalence of hypertension among the respondents was 20% (18.5% stage I HTN and 1.5% stage II HTN). Nearly 19.7% were current alcohol users, whereas around 29.1% were found to be current smokers.

**Table 2:** Sociodemographic profile of study participants (n=910).

Sociodemographic variables	Male	Female	Total (n=910)
Age-group			
18-29 years	79 (59.8%)	53 (40.2%)	132 (14.5%)
30-49 years	297 (67.6%)	142 (32.4%)	439 (48.3%)
50-59 years and above	172 (50.7%)	167 (49.3%)	339 (37.2%)
Mean±SD	38.72±11.6	42.06±11.6	40.0±11.7
Religion			
Hindu	447 (58.5%)	317 (41.5%)	764 (84.0%)
Non-Hindu	101 (69.2%)	45 (30.8%)	146 (16.0%)
Marital status			
Single	92 (55.7%)	73 (44.3%)	165 (18.1%)
Married	456 (66.4%)	231 (33.6%)	687 (75.5%)
Widowed	0 (0.0%)	45 (100.0%)	045 (5.0%)
Separated/divorced	0 (0.0%)	13 (100.0%)	13 (1.4%)
Education			
Primary	165 (56.3%)	128 (46.7%)	293 (32.1%)
Middle	76 (56.7%)	58 (43.3%)	134 (14.7%)
Matric	113 (69.3%)	50 (30.7%)	163 (18.0%)
SSC/intermediate	85 (83.3%)	17 (16.7%)	102 (11.2%)
Graduate	37 (86.0%)	06 (14.0%)	43 (4.8%)
Postgraduate/above	42 (80.8%)	10 (19.2%)	52 (5.7%)
Illiterate	30 (24.39%)	93 (75.61%)	123 (13.5%)
Occupation			
Government employee	94 (94.0%)	06 (6.0%)	100 (11.0%)
Non-government employee	91 (70.5%)	38 (29.5%)	129 (14.2%)
Homemaker	176 (41.4%)	249 (58.6%)	425 (46.7%)
Self-employed	165 (73.3%)	60 (26.7%)	225 (24.7%)
Student/retired/unemployed (able to work)	22 (71.0%)	09 (29.0%)	31 (3.4%)
Socio-economic status of the family (modified BG Prasad scale)			
Upper class	94 (80.3%)	23 (19.7%)	117 (12.9%)
Upper middle class	260 (53.5%)	226 (46.5%)	486 (53.4%)
Middle class	152 (72.4%)	58 (27.6%)	210 (23.0%)
Lower middle class	42 (75.0%)	14 (25.0%)	56 (6.2%)
Lower class	0 (0.0%)	41 (100.0%)	41 (4.5%)
Prevalence of current tobacco consumption	Yes	No	Total
Male	206 (37.6%)	342 (62.4%)	548 (60.2%)
Female	59 (16.3%)	303 (83.7%)	362 (39.8%)
Prevalence of current alcohol consumption	Yes	No	Total
Male	168 (30.7%)	380 (69.3%)	548 (60.2%)
Female	11 (3.0%)	351 (97.0%)	362 (39.8%)
Prevalence of hypertension (HTN)	Stage I	Stage II	Total
Male	136 (96.4%)	05 (3.6%)	141 (15.5%)
Female	33 (80.4%)	8 (19.6%)	41 (5.5%)

In the present study, those with random blood glucose level >200 g/dL were considered to have diabetes mellitus as per the operational definition. Out of 910 subjects, only 895 volunteered for RBS estimation, giving a response rate of 98.3%. Table 3 shows that, in RBS estimation, 55 subjects (6.2%) had an RBS level greater than 200 mg/dL. All 55 subjects were referred to the nearest PHC for further investigation and confirmation.

**Table 3:** Prevalence of diabetes mellitus among study subjects (n=895).

Diabetes mellitus	Frequency	Percentage
Present	55	6.2%
Absent	840	93.8%
Total	895	100%

The prevalence of diabetes among females was 8.7% which was comparatively higher than the prevalence among males, i.e., 4.5%. This difference was found to be statistically significant (p<0.05). The prevalence among the 50-59-year age group was 12% as compared to 2.32% in the 30-49-year age group and 3.87% in the 18-29-year age group. The difference was found to be statistically significant (p<0.001). The prevalence of DM was found to be significantly higher among individuals who were hypertensive (21.9%), overweight, obese (45.5%), and those with a family history of DM (38.1%), as shown in Table 4. No difference was found in prevalence by residence, social group, educational status, smoking, or alcohol use.

**Table 4:** Diabetes and associated risk factors among study population (n=55).

Variables	Diabetes prevalence	Chi-square value	p-Value
Gender		ꭓ^2^=6.01	p<0.05
Male	24 (4.5%)		
Female	31 (8.7%)		
Age group (years)		ꭓ^2^=31.59	p<0.001
18-29	5 (3.9%)		
30-49	10 (2.3%)		
50-59	40 (12.0%)		
Overweight and obesity		ꭓ^2^=26.58	p<0.001
Yes	45 (11.0%)		
No	10 (2.0%)		
Family history of diabetes		ꭓ^2^=9.66	p=0.001
Yes	21 (11.5%)		
No	34 (4.8%)		
Family history of raised cholesterol		ꭓ^2^=3.02	p=0.08
Yes	07 (11.9%)		
No	48 (5.8%)		
Family history of high blood pressure		ꭓ^2^=6.71	p=0.009
Yes	32 (8.9%)		
No	23 (4.3%)		

In multivariate logistic regression analysis, adults aged 50-59 years had 3.4-fold increased odds of diabetes compared to those aged 18-29 years (OR=3.40, 95% CI: 1.30-8.90, p=0.010) (Table 5). Individuals with overweight or obesity were at the highest risk, with nearly a sixfold increase in odds (OR: 5.80, 95% CI: 2.90-11.70, p<0.001). Surprisingly, a family history of diabetes was associated with significantly lower odds of having diabetes themselves (OR: 0.30, 95% CI: 0.20-0.70, p=0.001). This could be due to awareness and early lifestyle interventions. No significant association was found between a history of hypertension and the risk of diabetes. Although hypertension is a known comorbid condition, its effect may overlap with other included predictors like age and obesity.

**Table 5:** Adjusted odds ratios for diabetes (multivariate logistic regression).

Variables	Adjusted OR (95% CI)	p-Value
Age 30-49 vs. 18-29	0.60 (0.20, 1.70)	0.300
Age 50-59 vs. 18-29	3.40 (1.30, 8.90)	0.010
Primary education vs. illiteracy	0.50 (0.20, 1.20)	0.100
Secondary education vs. illiteracy	0.40 (0.20, 0.90)	0.040
Higher education vs. illiteracy	0.70 (0.20, 1.90)	0.500
Hypertension (yes vs. no)	0.80 (0.40, 1.50)	0.500
Overweight/obese (yes vs. no)	5.80 (2.90, 11.70)	0.001
Family history of diabetes (yes vs. no)	0.30 (0.20, 0.70)	0.001

## Discussion

Owing to the morbidity and mortality rates attributable to type 2 diabetes regionally and globally, early detection remains the health-promoting practice of choice. In this cross-sectional survey, the prevalence of diabetes mellitus was 6.2%. The prevalence in the present study is similar to estimates from previous regional studies by Thankappan et al., Ghobadzadeh et al., Thakur et al., and Maimela et al. [10-13]. It was lower than the findings of the ICMR-INDIAB study, which reported 9.5% prevalence in rural areas, but comparable to the national data from NFHS-5 (2019-21) [14]. This difference could be attributed to the difference in methodologies. In the present study and the NFHS-5, random blood glucose estimation was performed, whereas the ICMR-INDIAB study utilized both fasting and post-prandial blood glucose levels to determine diabetes prevalence [14].

Our finding of higher diabetes prevalence among women aligns with a WHO STEPS-based Delhi survey, which also observed elevated rates in females [15], but diverges from NFHS-5 findings, which noted higher rates among men. This could be due to hormonal factors involved in insulin resistance, as well as similarities in lifestyle and physical activity among women in Delhi-NCR, which contribute to their higher risk of diabetes. Moreover, prevalence rose significantly with advancing age, as noted in a consistent trend in the Punjab STEPS study and U.S. studies by Cowie et al. [16,17]. In concordance with the present study, various other studies, Ramachandran et al., Howard et al., Ajay et al., Bharati et al., and Bhalerao et al., have reported advancing age as an important risk factor for diabetes [18-22]. The role of age as a non-modifiable risk factor for diabetes is well known.

Various studies have reported that alcohol consumption was associated with an increased prevalence of diabetes. However, in the present study, no significant difference was found in the prevalence of diabetes among alcohol users. Bhalerao et al. conducted a study in North Karnataka and reported alcohol as a significant factor associated with diabetes [22]. Shah and Afzal also reported a positive association between alcohol and diabetes [23]. This is probably due to the development of insulin resistance, which is a key factor in the pathogenesis of type 2 diabetes mellitus among heavy alcohol drinkers, and this has been shown by some studies to be mediated by increased obesity, especially abdominal obesity. Literature by Howard et al. showed varied associations between alcohol consumption and increased risk of diabetes [19]. But contrary to this, Wei et al. reported the protective effect of alcohol on blood glucose levels [24].

A higher prevalence of raised blood glucose was observed among hypertensive individuals in the present study; however, the finding was not statistically significant. Hypertension may not have emerged as an independent predictor due to mediated pathways through obesity or age-related metabolic dysregulation. However, studies by Thankappan et al., Ajay et al., Kokiwar et al., Aravindalochanan et al., and Midha et al. have reported a significant association between hypertension and diabetes [10,20,25-28]. The results of this study indicate that advancing age and obesity were strongly associated with diabetes. Notably, the present study revealed an inverse association between the participants' family history of diabetes and their own diabetes risk. This could be due to the fact that individuals who are aware of a familial predisposition may engage in risk-mitigating behaviors, such as earlier screening, healthier diets, or lifestyle changes, which can significantly reduce diabetes incidence in high-risk populations.

This study had a few limitations. As this is a cross-sectional study, it prevents us from drawing causal inferences. Additionally, despite multivariable adjustment, confounding, and potential selection or information bias could still influence the results. However, the large sample size and use of calibrated instruments minimized information bias and improved precision. Also, measurement of blood glucose was performed using a glucometer device instead of venous blood glucose estimation due to logistical constraints, although this was mitigated by routine calibration checks. By employing the standardized and pretested WHO STEPS instrument, data on behavioral, anthropometric, and biochemical risk factors were reliably captured, enhancing comparability across surveys.

## Conclusions

The present study provides reliable and recent epidemiological information on the high burden of diabetes mellitus among the adult population in a representative rural Indian population, highlighting the need for immediate attention. Community-based interventions, including blood screening and health education, would be impactful in similar demographic settings. However, efforts must be made towards identifying the large pool of undiagnosed cases of diabetes and offering early treatment in order to avoid complications. There is a need for a collective approach involving individuals, healthcare providers, and policymakers to curb the silent epidemic.
